# Progress in Drug Delivery Systems Based on Nanoparticles for Improved Glioblastoma Therapy: Addressing Challenges and Investigating Opportunities

**DOI:** 10.3390/cancers17040701

**Published:** 2025-02-19

**Authors:** Md Ataur Rahman, Maroua Jalouli, Mahesh Kumar Yadab, Mohammed Al-Zharani

**Affiliations:** 1 Department of Oncology, Karmanos Cancer Institute, Wayne State University, Detroit, MI 48201, USA; gm7043@wayne.edu; 2 Department of Biology, College of Science, Imam Mohammad Ibn Saud Islamic University (IMSIU), Riyadh 11623, Saudi Arabia; mejalouli@imamu.edu.sa (M.J.); mmylzahrani@imamu.edu.sa (M.A.-Z.)

**Keywords:** nanoparticles, drug delivery, glioblastoma, nanomedicine, targeted therapy

## Abstract

Glioblastoma multiforme (GBM) is a highly malignant brain tumor that has a bleak outlook despite existing treatments such as surgery, radiation, and chemotherapy. The utilization of nanoparticles for drug delivery presents a promising method by which to improve the effectiveness of treatment while reducing the harmful effects on the entire body. This review examines the application of nanoparticles in the treatment of GBM, focusing on different types of nanoparticles, including lipid-based, polymeric, metallic, and those under development. Every variety is analyzed for its distinct characteristics and therapeutic capacity. Lipid-based nanoparticles, such as liposomes and solid lipid nanoparticles, enhance the transport of medicines that are not soluble in water and have shown considerable potential in preclinical investigations. Polymeric nanoparticles have benefits in terms of controlled release and targeted distribution, whereas metallic nanoparticles have potential in both therapy and imaging. In the current review we would like to emphasize the ways in which nanoparticles improve medicine delivery, specifically by enhancing penetration of the blood–brain barrier (BBB), targeting tumors, and enabling controlled release. Additionally, we also discuss current preclinical and clinical discoveries, highlighting both achievements and obstacles in the process of converting these technologies into effective treatments for GBM. This study offers a thorough examination of the present status and prospects of nanoparticles in the treatment of GBM.

## Simple Summary

Glioblastoma multiforme (GBM) is an aggressive brain tumor with limited treatment options. Nanoparticles offer a promising strategy to enhance drug delivery while minimizing side effects. This review explores different types of nanoparticles, including lipid-based, polymeric, and metallic, and their role in improving GBM treatment. These nanoparticles help transport drugs across the blood–brain barrier, target tumors more effectively, and enable controlled drug release. We discuss recent preclinical and clinical findings, highlighting both progress and challenges in translating these technologies into successful GBM therapies.

## 1. Introduction

Glioblastoma multiforme (GBM) is a malignant and deadly kind of brain cancer [1]. It is known for its fast growth, widespread infiltration into nearby brain tissue, and the presence of diverse cell types [2]. GBM constitutes around 15% of all primary brain tumors and over 50% of all malignant brain tumors [3,4]. Despite the progress made in medical technology and therapeutic approaches, the outlook for GBM is still quite poor, with a median survival rate of 12 to 15 months after diagnosis [5]. The five-year survival rate is significantly poor, approximately 4–7%, emphasizing the urgent requirement for more efficient treatments [6]. GBM is being treated using a multimodal approach that combines surgical resection, radiation therapy, and chemotherapy [7]. The primary objective of surgery is to exercise the tumor mass to the greatest extent feasible [8]. Nevertheless, achieving total resection is sometimes unattainable due to the tumor’s invasive characteristics and its proximity to critical brain structures [9]. The presence of this remaining tumor frequently results in the reappearance of the disease [10]. Radiation therapy, a treatment that employs powerful beams of energy to specifically target and eliminate cancer cells, is commonly delivered after surgery [11]. However, its effectiveness is hindered by the risk of harming normal brain tissue, resulting in cognitive and neurological complications [12].

GBM is typically treated with chemotherapy, namely using the alkylating drug temozolomide (TMZ), which is considered the standard systemic treatment [13,14]. Although TMZ has demonstrated some effectiveness in prolonging survival, its effectiveness is restricted by the emergence of resistance in GBM cells and the inability of numerous chemotherapeutic drugs to penetrate the blood–brain barrier (BBB) [15]. The BBB is a discerning and partially permeable boundary that shields the brain from harmful substances and disease-causing microorganisms [16]. However, it also limits the transportation of numerous medicinal substances [17]. This is a substantial obstacle in the treatment of GBM, as it greatly restricts the number of medications that can be delivered to the tumor site [18]. Considering these constraints, there is a pressing requirement for innovative therapeutic strategies that can precisely target and treat GBM. Recent breakthroughs in nanotechnology present a hopeful opportunity for tackling the difficulties presented by GBM, specifically in surmounting the barriers associated with drug transportation and the BBB.

Nanoparticles have become a potent instrument in cancer treatment, providing distinctive characteristics that render them well-suited for drug delivery systems [19]. Nanoparticles are often described as particles with sizes ranging from 1 to 100 nanometers [20] and which possess the capacity to transport and administer therapeutic substances directly to cancer cells [21]. Nanoparticles possess advantageous characteristics such as their diminutive size, significant surface area-to-volume ratio, and adjustable surface qualities [22]. These attributes enable accurate drug targeting and regulated drug release, rendering them an appealing choice for the treatment of malignancies such as GBM [23]. The presence of the BBB poses a major obstacle in the treatment of brain malignancies, such as GBM [18]. The BBB consists of closely interconnected endothelial cells that form the lining of the brain’s blood arteries [24]. Its main function is to restrict the passage of most medicines and therapeutic agents into the brain [25]. Nanoparticles have demonstrated the capacity to traverse the BBB by different routes, including receptor-mediated transcytosis, adsorption-mediated transcytosis, and passive diffusion [26]. Through the utilization of these routes, nanoparticles could efficiently transport medications directly to the location of the brain tumor [27]. This has the potential to enhance the amount of the therapeutic substance at the tumor site while reducing the overall toxicity to the body [21].

Nanoparticles have numerous potential benefits in drug delivery for GBM [28,29]. Firstly, they can be manipulated to enhance the solubility and stability of medications that have low solubility, hence improving their bioavailability [28,30]. Furthermore, nanoparticles have the capability to be modified with ligands or antibodies that selectively bind to cancer cells, thereby minimizing unintended consequences and enhancing therapeutic efficacy [23]. In addition, nanoparticles can be engineered to selectively release their therapeutic payload in response to stimuli, such as alterations in pH or enzyme activity inside the tumor microenvironment [31,32]. This enables precise and continuous drug delivery [33]. Furthermore, nanoparticles can transport many therapeutic drugs simultaneously, which allows for combination therapy that can target various pathways implicated in the progression and resistance of GBM [34]. This strategy has the capacity to surpass the restrictions of conventional monotherapies and enhance overall treatment outcomes [35]. In this review we would like to draw attention to the utilization of nanoparticles in cancer therapy, which shows great potential as a technique to tackle the difficulties associated with GBM treatment. Therefore, nanoparticles have the potential to transform the management of GBM and enhance patient outcomes by overcoming the limits of traditional therapy, especially in delivering drugs across the BBB.

## 2. Types of Nanoparticles Used in GBM Therapy

**Figure 1:**
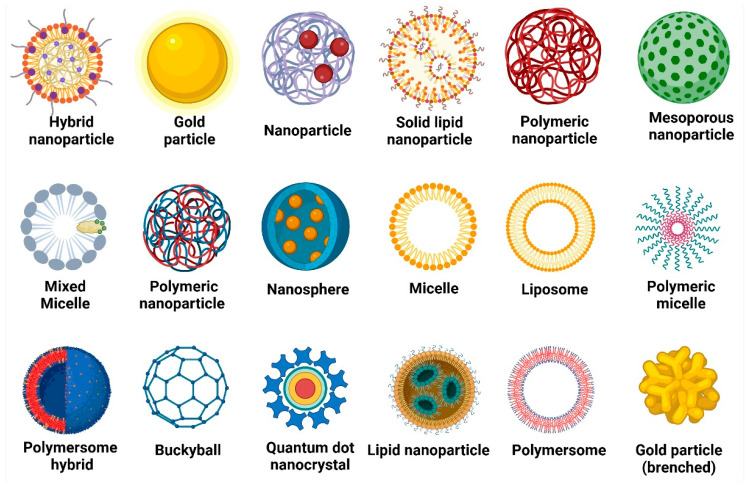
Types of nanoparticles that make up pharmaceutical delivery systems in glioblastoma. Gold nanoparticles and gold particles (branched) have been widely explored for photothermal therapy, leveraging their ability to convert light into heat and selectively destroy tumor cells. Hybrid nanoparticles integrate multiple functional materials, enhancing drug delivery efficiency and imaging capabilities. Nanoparticles, in general, serve as carriers for chemotherapeutic agents, improving their solubility and tumor targeting. Solid lipid nanoparticles and lipid nanoparticles offer biocompatibility and controlled drug release, effectively delivering temozolomide, the standard chemotherapy for GBM. Polymeric nanoparticles, including nanospheres, enable sustained drug release, reducing systemic toxicity. Mesoporous nanoparticles provide high drug-loading capacity, optimizing the delivery of small-molecule inhibitors. Mixed micelles and micelles enhance the solubility and bioavailability of hydrophobic drugs, facilitating their penetration through the BBB. Polymeric micelles improve drug stability, while polymersomes and polymersome hybrids offer advanced encapsulation of both hydrophilic and hydrophobic therapeutics, ensuring controlled release within the tumor microenvironment. Liposomes, with their phospholipid bilayer, encapsulate chemotherapeutic drugs and RNA-based therapies, protecting them from enzymatic degradation. Buckyballs (fullerenes) possess antioxidant properties that may counteract oxidative stress-induced tumor progression. Quantum dot nanocrystals enable precise imaging, aiding in tumor detection and surgical guidance. The figure was created using the BioRender.com online commercial platform.

Nanoparticles have become useful implements in GBM therapy owing to their capacity to circumvent the blood–brain barrier, transport medications directly to the tumor, and augment the effectiveness of treatment [12]. The various nanoparticles used in pharmaceutical delivery systems, including polymeric nanoparticles, liposomes, dendrimers, metallic nanoparticles, and lipid-based nanoparticles are presented in Figure 1. Each type is analyzed for its unique structure and potential application in targeted therapies, particularly for glioblastoma and other cancer treatments. These diverse nanoparticles hold significant potential in GBM treatment, revolutionizing drug delivery, imaging, and therapeutic strategies for improved patient outcomes.

### 2.1. Lipid-Based Nanoparticles

#### 2.1.1. Liposomes

Liposomes are spherical structures made up of a double layer of phospholipids and which are capable of enclosing pharmaceuticals that are both water soluble and oil soluble [36]. Liposomes have been thoroughly researched for their biocompatibility and their potential use in drug delivery for GBM therapy [37,38]. Liposomes have the capacity to entrap hydrophobic medications within their lipid bilayer, shielding them from degradation and improving their bioavailability [39]. Additionally, they possess the benefit of passive targeting to the tumor site through the enhanced permeability and retention (EPR) effect [40]. Several preclinical investigations have shown that liposome-encapsulated medicines are effective in models of GBM [41]. For instance, the use of liposomal formulations of doxorubicin and temozolomide has demonstrated encouraging outcomes in enhancing the transportation of drugs to the brain tumor while minimizing the overall toxicity to the body [42].

#### 2.1.2. Solid Lipid Nanoparticles (SLNs)

Solid lipid nanoparticles (SLNs) are a type of lipid-based nanoparticle that has numerous benefits in the field of medication delivery [43]. SLNs consist of solid lipids, which enhance their stability compared with conventional liposomes [43]. In addition, they can enclose hydrophobic medications, thus enhancing their solubility and stability [44]. Sentinel lymph node nanoparticles (SLNs) have demonstrated promise in transporting chemotherapeutic drugs to GBM tumors, as research has indicated increased drug concentration at the tumor location and greater treatment results [45]. For example, SLNs containing paclitaxel have shown enhanced ability to kill GBM cells and decreased tumor growth in animal experiments [46].

#### 2.1.3. Benefits of Encapsulating Hydrophobic Drugs

**Figure 2:**
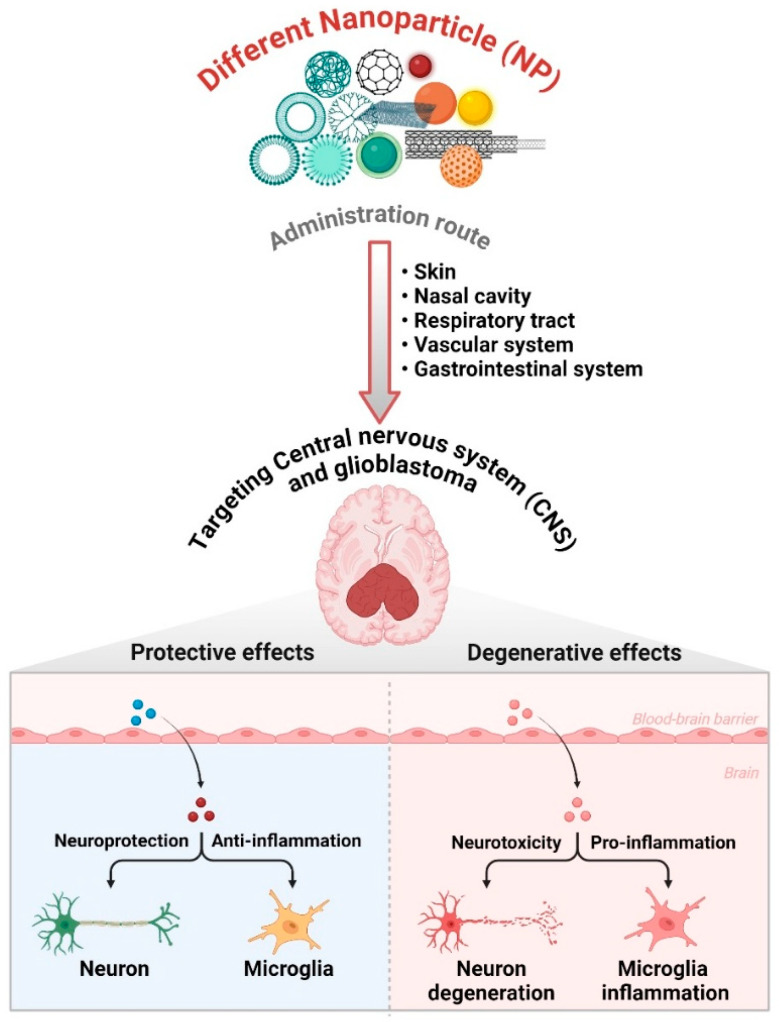
Effects of different nanoparticles on the central nervous system and glioblastoma. Nanoparticles (NPs) are administered into the central nervous system (CNS), including the gastrointestinal tract, respiratory system, nasal cavity, and skin. NPs cross the BBB to specifically target various brain cells such as neurons, microglia, and astrocytes. The impact of these nanoparticles can be either protective or degenerative, depending upon their characteristics and interactions within the central nervous system. The function of NPs in therapeutic approaches, especially in the treatment of glioblastoma, is in their ability to regulate the advancement of the disease and the reactions of cells in the CNS environment. The figure was created and modified using the BioRender.com online commercial platform.

Lipid-based nanoparticles, such as liposomes and SLNs, are beneficial for encapsulating hydrophobic medicines because of their lipid bilayer structure [47]. Hydrophobic medications frequently experience inadequate solubility and absorption, which restricts their therapeutic efficacy [48]. Through the process of encapsulating these medications in lipid-based nanoparticles, their solubility and stability experience a substantial increase, resulting in better drug delivery to the GBM tumor site [18]. In addition, lipid-based nanoparticles can be altered with ligands or antibodies to actively target specific areas, thus increasing their precision and effectiveness in treating GBM [18] (Figure 2).

#### 2.1.4. Examples of Preclinical and Clinical Studies

Multiple preclinical and clinical investigations have examined the application of lipid-based nanoparticles in the treatment GBM [18]. During a preclinical investigation, liposomal temozolomide demonstrated enhanced drug accumulation in the brain and enhanced survival in a mouse model of GBM [18]. A separate study provided evidence of the effectiveness of SLNs containing curcumin in decreasing the size of tumors and extending the lifespan of rats with GBM [49]. From a clinical perspective, liposomal formulations such as Doxil (liposomal doxorubicin) have been studied in patients with GBM, demonstrating encouraging outcomes in terms of both safety and effectiveness [50]. Additional clinical trials are necessary to definitively determine the therapeutic efficacy of lipid-based nanoparticles in the treatment of GBM.

### 2.2. Polymeric Nanoparticles

#### 2.2.1. Polymers Like PLGA and Chitosan

Polymeric nanoparticles consist of biodegradable polymers, which make them appropriate for the purpose of regulated and prolonged drug release [51]. Poly(lactic-co-glycolic acid) (PLGA) and chitosan are commonly utilized polymers in GBM therapy [52]. PLGA is a highly utilized polymer because of its compatibility with living organisms, its ability to break down naturally, and its capacity to enclose a diverse array of medications [53]. Chitosan, a natural polymer obtained from chitin, is utilized in nanoparticle formulation due to its biocompatibility and capacity to improve drug absorption through biological barriers [54].

#### 2.2.2. Properties of Biodegradability and Controlled Drug Release

A significant benefit of polymeric nanoparticles is their capacity to biodegrade, resulting in the progressive conversion of the nanoparticles into harmless substances [55]. The combination of this characteristic, along with the capacity to regulate the release of drugs, renders polymeric nanoparticles highly suitable for long-lasting drug administration in GBM treatment [56]. Through the process of enclosing pharmaceuticals within polymeric nanoparticles, it becomes feasible to achieve a sustained release of the therapeutic agent [57]. This allows for the maintenance of drug levels within the desired therapeutic range for an extended duration [58]. The property of controlled release can enhance the effectiveness of the treatment and decrease the frequency of drug administration [59].

#### 2.2.3. Applications for the Treatment of GBM

Extensive research has been conducted on polymeric nanoparticles for their use in treating GBM [10]. PLGA nanoparticles have been utilized to transport temozolomide, the established chemotherapeutic agent for GBM, leading to amplified drug delivery and enhanced therapeutic results [60]. Chitosan-based nanoparticles have been investigated for use in the delivery of different anticancer medicines, such as doxorubicin and paclitaxel, to GBM tumors [61]. These investigations have shown that polymeric nanoparticles could boost drug delivery, increase the effectiveness of therapy, and overcome the difficulties presented by the BBB in the treatment of glioblastoma.

### 2.3. Metallic Nanoparticles

#### 2.3.1. Gold Nanoparticles

Gold nanoparticles (AuNPs) are being widely studied for their distinct optical and electrical characteristics, making them highly promising for treating GBM [62]. AuNPs can be readily modified with different ligands, antibodies, or medicines, facilitating specific delivery to GBM cells [63]. AuNPs, due to their small size and surface features, can traverse the BBB and mass within the tumor tissue [64]. AuNPs have drug delivery capabilities and can also be utilized for photothermal therapy, wherein they absorb light and transform it into heat, thus efficiently eradicating cancer cells [65]. Multiple preclinical investigations have demonstrated that AuNPs carrying chemotherapeutic medicines or modified with targeting ligands can augment the delivery of drugs to GBM tumors and enhance treatment results [66].

#### 2.3.2. Nanoparticles of Iron Oxide

Nanoparticles of iron oxide (IONPs) offer a promising approach for targeted GBM therapy due to their excellent biocompatibility, magnetic properties, and functionalization potential [67]. By conjugating targeting ligands, antibodies, or peptides to the surface of IONPs, it is feasible to achieve precise binding to GBM cells, minimizing off-target effects and enhancing therapeutic efficacy [68]. For example, iron oxide nanoparticles functionalized with anti-EGFR antibodies have demonstrated specific targeting capabilities against GBM cells overexpressing the EGFR receptor [69]. Additionally, their magnetic properties allow for magnetic resonance imaging (MRI) contrast enhancement, facilitating both the diagnosis and the real-time monitoring of treatment response [70]. Research has shown that IONPs can transport medications such as temozolomide and doxorubicin [71] to GBM tumors, resulting in better treatment results compared with traditional methods [72].

An important benefit of metallic nanoparticles is their capacity to be functionalized for precise therapeutic targeting [73]. By conjugating targeting ligands, antibodies, or peptides to the surface of metallic nanoparticles, it is feasible to obtain precise binding to GBM cells, reducing off-target effects and improving therapeutic effectiveness [74]. For instance, AuNPs that have been modified with anti-EGFR antibodies have demonstrated the ability to specifically target GBM cells that have an excessive expression of the EGFR receptor [65]. This targeted approach enhances the delivery of drugs and effectively kills tumor cells [75]. Moreover, the utilization of IONPs that have been modified with RGD peptides has demonstrated their increased ability to specifically target GBM cells and has resulted in improved treatment results in preclinical models [76].

#### 2.3.3. Imaging and Therapeutic Capabilities

Metallic nanoparticles, specifically AuNPs and IONPs, provide two-fold capabilities for both imaging and therapy [77]. AuNPs have the potential to be employed in drug delivery and photothermal therapy, whereas IONPs can be utilized for drug delivery and imaging based on MRI [78]. This theragnostic technique enables simultaneous monitoring of medication distribution and treatment response in real time, offering useful insights for optimizing therapy [79]. Furthermore, the integration of imaging and therapy within a single nanoparticle platform can augment the overall efficacy of GBM treatment, resulting in improved patient outcomes [10].

### 2.4. Other Emerging Nanoparticles

#### 2.4.1. Carbon Nanotubes

Carbon nanotubes (CNTs) are nanostructures that have a cylindrical shape and are made up of graphene sheets that are rolled into tubes [80]. CNTs possess distinctive characteristics, such as a large surface area, electrical conductivity, and the capability to permeate biological membranes [81]. The characteristics of CNTs make them highly promising candidates for medication administration in the therapy of GBM [82]. CNTs can be modified with different medications, ligands that specifically bind to target molecules, or compounds used for medical imaging [83]. This enables precise delivery of drugs to specific locations and visualization of GBM tumors [84]. Nevertheless, the therapeutic use of CNTs has been restricted due to concerns over their toxicity and biocompatibility [85]. Consequently, additional research is necessary to tackle these obstacles.

#### 2.4.2. Dendrimers

Dendrimers are complex nanostructures characterized by a central core and several terminal groups, like a massively branching tree [86]. Dendrimers possess a distinctive architecture that enables them to encapsulate pharmaceuticals within their internal cavities and attach targeted ligands or imaging agents to their surface [87]. Dendrimers have demonstrated promise in transporting several therapeutic agents to GBM tumors, encompassing chemotherapeutic medicines, nucleic acids, and imaging agents [88]. Dendrimers show promise as a valuable tool in GBM therapy due to their capacity to improve drug solubility, shield medicines from degradation, and selectively target certain cells [89]. Nevertheless, the intricate nature of dendrimer manufacturing and the necessity to resolve potential toxicity concerns must be addressed prior to their widespread application in clinical environments [90].

#### 2.4.3. Quantum Dots

Quantum dots (QDs) are nanocrystals made of semiconductors that possess distinct optical characteristics, such as fluorescence that vary with their size [91]. QDs have been investigated for their potential use in both imaging and drug delivery in the treatment of GBM [92]. QDs, with their compact dimensions and adjustable surface characteristics, can be combined with medications, targeting molecules, or imaging substances, enabling the simultaneous application of imaging and treatment [93]. QDs have the benefit of exhibiting a high level of fluorescence intensity, which allows for the detection of even minute amounts of the nanoparticle within the tumor [94]. Nevertheless, there are apprehensions regarding the toxicity of QDs [95].

## 3. Mechanisms of Nanoparticle-Mediated Drug Delivery in GBM Therapy

Nanoparticles (NPs) have become a favorable option for transporting drugs due to their potential to deliver drugs to specific locations, regulate the release of drugs, and overcome obstacles in the body [96]. This section specifically inspects the methods by which nanoparticles are used to deliver drugs, with a special emphasis on efforts to improve their ability to pass through the BBB, target tumors, and release and deliver drugs inside cells. Table 1 offers an overview of various nanoparticles employed in glioblastoma therapy for drug delivery, encompassing their nomenclature, dimensions, delivery mechanisms, and molecular functions.

### 3.1. Targeting the Blood–Brain Barrier (BBB)

**Figure 3:**
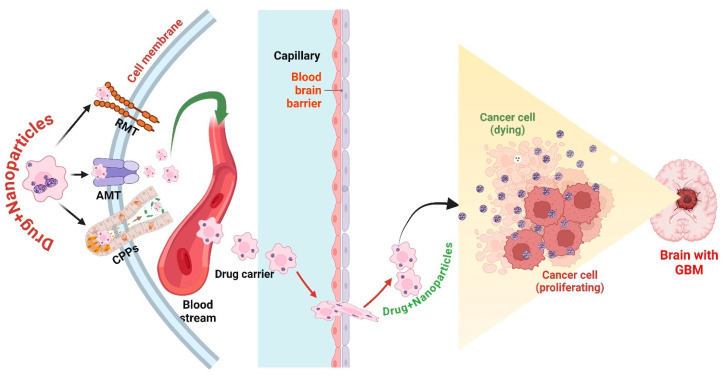
Several key mechanisms are employed by nanoparticles to traverse the BBB and efficiently deliver drugs to GBM cells. Receptor-mediated transcytosis (RMT): Nanoparticles modified with ligands that target specific receptors on the endothelial cells of the BBB, such as transferrin, insulin, or low-density lipoprotein receptors. Ligand binding promotes nanoparticle internalization and transcytosis, facilitating drug delivery to the brain parenchyma and GBM cells. Adsorptive-mediated transport (AMT): Demonstration of cationic nanoparticles employing electrostatic interactions with negatively charged membrane constituents of BBB endothelial cells. This process improves nanoparticle absorption and transcytosis, facilitating the effective delivery of therapeutic drugs across the BBB for GBM treatment. Cell-penetrating peptides (CPPs): Visualization of nanoparticles coupled with CPPs, like TAT or penetratin, to improve BBB penetration. These peptides enable direct nanoparticle translocation across endothelial cells, facilitating efficient medication delivery to GBM while reducing systemic exposure. The figure was created and modified using the BioRender.com online commercial platform.

The BBB is a highly discerning barrier that shields the brain from potentially detrimental substances while controlling the movement of vital molecules [116]. Nevertheless, this defensive role presents a notable obstacle for administering drugs to the brain, especially regarding the treatment of GBM [117]. Nanoparticles provide a solution to this difficulty by employing different tactics that improve their ability to pass the BBB. Functionalization of nanoparticles with ligands that selectively bind to receptors such as transferrin or lactoferrin on the BBB can be achieved by receptor-mediated endocytosis to enhance cargo transportation. Nanoparticle-mediated drug delivery enhances the precision and efficacy of GBM treatment while minimizing systemic toxicity and unintended side effects. Additionally, advancements in nanoparticle engineering, including stimuli-responsive, targeted, and biodegradable nanocarriers, continue to improve therapeutic outcomes in glioblastoma therapy (Figure 3).

#### 3.1.1. Methods to Improve Nanoparticle Permeation

An important approach to improving the ability of nanoparticles to pass the BBB is to carefully adjust the size, shape, and surface charge of the nanoparticles. Nanoparticles with smaller sizes, typically ranging from 1 to 100 nm, have a higher likelihood of crossing the BBB because they can effectively avoid the reticuloendothelial system (RES) and pass through the tight junctions of the BBB [118]. Additionally, the morphology of nanoparticles can impact their interaction with endothelial cells [119]. For instance, studies have demonstrated that rod-shaped nanoparticles exhibit greater endothelial uptake in comparison to spherical nanoparticles. Surface charge is an important and influential component [120]. Nanoparticles with a positive charge have a more advantageous interaction with the negatively charged endothelial cells of the BBB, which enhances their cellular absorption [121]. Nevertheless, an overabundance of positive charge can result in cellular toxicity and non-selective attachment, highlighting the need for a well-balanced strategy in the creation of nanoparticles [76].

#### 3.1.2. Surface Modification Using Targeting Ligands

Coating nanoparticles with specific ligands is a useful approach to improve the ability of the particles to cross the BBB and deliver drugs directly to glioblastoma cells [122]. Transferrin, lactoferrin, and insulin are ligands that have been thoroughly investigated for their capacity to attach to receptors that are excessively expressed on the endothelial cells of the BBB [123]. After the ligand attaches to its receptor, the nanoparticle can be taken inside the cell through receptor-mediated transcytosis, which helps it move across the BBB [124]. Another method entails utilizing cell-penetrating peptides (CPPs), which are brief sequences of amino acids capable of traversing cellular membranes [125]. Cationic CPPs can be attached to the surface of nanoparticles to increase their ability to pass through the BBB [125]. An example of this is the utilization of the TAT peptide, which is derived from the HIV-1 virus, to effectively enhance the ability of different nanoparticle formulations to penetrate the BBB [126].

### 3.2. Tumor Targeting Mechanisms

**Figure 4:**
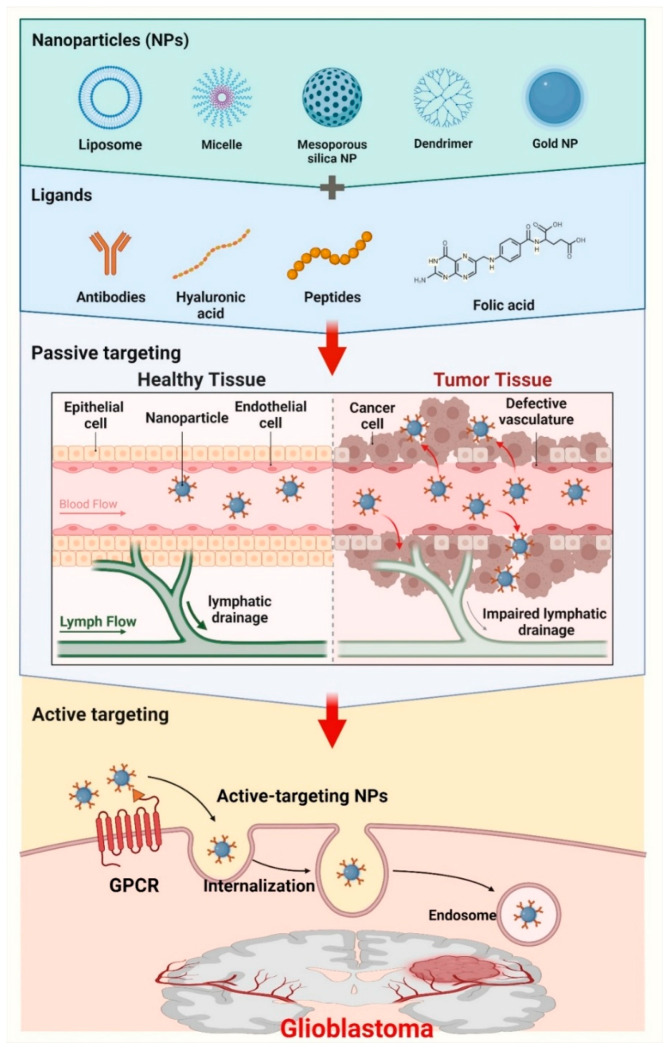
GBM targeting through both passive and active strategies. GBM passive targeting techniques use enhanced permeability and retention (EPR) phenomenon, where nanoparticles concentrate in tumor tissue due to solid tumors’ leaky vasculature. Nanoparticles leave the bloodstream and aggregate in the tumor’s interstitial spaces to deliver drugs locally. Passive targeting may not be enough for optimum medication administration due to tumor blood vascular heterogeneity and high interstitial pressure. Nanoparticle-mediated medication delivery for GBM can be improved by combining the EPR effect with additional targeted methods. Active targeting enhances drug selectivity and uptake by altering nanoparticles with ligands like the RGD peptide, which binds to the overexpressed αvβ3 integrin on GBM cells. Magnetic targeting, another active targeting strategy, uses magnetic nanoparticles guided by a magnetic field to deliver drugs locally and reduce off-target effects, increasing GBM treatment outcomes. GPCR: G protein-coupled receptor. The figure was modified using the BioRender.com online commercial platform.

Precise localization of the tumor site is essential for optimizing the efficacy of medication delivery systems utilizing nanoparticles. Tumor targeting can be accomplished by passive and active targeting techniques, each providing unique benefits in improving medication delivery to GBM cells (Figure 4).

#### 3.2.1. Passive Targeting: Exploiting Enhanced Permeability and Retention (EPR) Effect

Passive targeting utilizes the enhanced permeability and retention (EPR) effect, which is a characteristic observed in solid tumors, including GBM [127]. The EPR effect is defined by the atypical vasculature of tumors, which enables nanoparticles to selectively collect within the tumor tissue [128]. Tumors have blood arteries that are prone to leakage, allowing nanoparticles to exit the bloodstream and accumulate in the space between cells in the tumor [129]. This enables the direct delivery of therapeutic substances to the tumor site [130]. Nevertheless, relying solely on the EPR effect may not be adequate for achieving optimal drug administration because of the diverse composition of tumor blood vessels and the elevated interstitial pressure within tumors. To address these constraints, the EPR effect is frequently integrated with other targeting approaches.

#### 3.2.2. Active Targeting: The Role of Ligand-Receptor Interactions and Magnetic Targeting

Active targeting refers to the process of modifying nanoparticles by attaching ligands that have a specific affinity for receptors that are excessively expressed on the outer surface of GBM cells [131]. These ligands may consist of antibodies, peptides, or tiny compounds that could identify tumor-specific antigens or receptors [132]. As an illustration, when nanoparticles are combined with the peptide RGD, they can specifically bind to the αvβ3 integrin, which is a receptor that is excessively produced in GBM cells. This interaction improves the specificity and absorption of the therapeutic drugs [133]. Magnetic targeting is a method of active targeting that involves the use of magnetic nanoparticles along with an external magnetic field [134]. Through the application of a magnetic field at the location of the tumor, it is possible to guide and concentrate magnetic nanoparticles specifically within the tumor. This enables the targeted administration of medication and minimizes the adverse effects on the rest of the body [135].

#### 3.2.3. Nanoparticles That Can React to Stimuli

Stimuli-responsive nanoparticles provide a sophisticated approach to target tumors by reacting to specific stimuli in the tumor microenvironment (TME), such as pH, temperature, or enzyme activity [136]. For example, the acidic conditions found in tumors can be utilized to activate the release of medications from nanoparticles that are sensitive to changes in pH [33]. These nanoparticles maintain stability at a pH level found in the body, but they experience changes in structure in the acidic TME, resulting in the release of the drugs they carry [137]. Temperature-sensitive nanoparticles can be engineered to release medications when exposed to hyperthermia, a treatment method commonly employed alongside radiotherapy or chemotherapy. Enzyme-responsive nanoparticles are a type of nanoparticles that can be triggered to release drugs by certain enzymes found in the TME by the cleavage of bonds in their structure [138].

### 3.3. Drug Release and Intracellular Delivery

The efficacy of nanoparticle-facilitated medication administration relies not only on the accurate targeting of GBM cells but also on the precise release of therapeutic chemicals and their intracellular delivery.

#### 3.3.1. Controlled and Sustained Drug Release Mechanisms

Nanoparticle-mediated drug delivery systems require controlled and sustained drug release as a crucial characteristic [37]. One way to accomplish this is by using biodegradable polymers or lipids that encapsulate the medication and gradually break down, resulting in a controlled release of the drug [139]. Modulating the release rate can be achieved by modifying either the polymer composition or the physicochemical characteristics of the nanoparticles, such as their size and surface charge [140]. Sustained release of medication guarantees a sustained therapeutic impact, hence decreasing the necessity for frequent administration and minimizing the risk of systemic toxicity. Furthermore, the use of controlled release mechanisms can effectively regulate medication concentrations within the desired therapeutic range, thus optimizing the medicine’s effectiveness while reducing any potential adverse effects.

#### 3.3.2. Endocytosis and Intracellular Trafficking

After nanoparticles have arrived at the tumor location, they need to be taken up by the GBM cells to release the medicine they carry [141]. Endocytosis is the main process by which nanoparticles are taken up by cells, and clathrin-mediated endocytosis is the most frequently observed method [142]. After being taken up by cells, nanoparticles are transported through endosomes and lysosomes, where the acidic conditions can stimulate the medicine to be released [143]. Nanoparticles can be designed to evade the endosomal pathway, therefore reducing breakdown in lysosomes and improving the intracellular transport of the medicine [144]. This can be accomplished by utilizing pH-sensitive compounds that breach the endosomal membrane, enabling the nanoparticles to discharge their cargo straight into the cytoplasm [145].

#### 3.3.3. Addressing Drug Resistance Mechanisms

Drug resistance poses a substantial barrier in the management of GBM, frequently resulting in the ineffectiveness of treatment [146]. Nanoparticles can be engineered to surmount drug resistance by simultaneously delivering numerous medicines that target distinct pathways or by impeding drug efflux pumps [147]. For instance, nanoparticles can be filled with chemotherapeutic medications and inhibitors of efflux pumps like P-glycoprotein (P-gp), which increases the number of pharmaceuticals inside cells and enhances their effectiveness in treatment [148]. In addition, nanoparticles can be modified with siRNA or miRNA to inhibit the expression of genes linked to drug resistance, hence increasing the responsiveness of GBM cells to therapy [149].

## 4. Preclinical and Clinical Studies of Nanoparticle-Mediated Drug Delivery for the Treatment of Glioblastoma Therapy

Although nanoparticle-mediated drug delivery shows potential for treating GBM, further research is needed to address the obstacles in transferring preclinical achievements into clinical practice. The outcome of the ongoing clinical trials and regulatory activities will be pivotal in shaping the future of nanoparticles in GBM therapy. Below is a concise summary of nanoparticles that have been recently utilized in the treatment of glioblastoma, providing information on their respective names, sizes, and molecular mechanisms as observed in both preclinical and clinical studies (Table 2).

### 4.1. Preclinical Efficacy of Nanoparticles in GBM Models

Experiments conducted on GBM cell lines in a laboratory setting have shown great potential in using nanoparticles for the purpose of delivering drugs. Typically, these investigations entail using well-known GBM cell lines, such as U87, U251, and T98G, to assess the harmful effects of chemotherapeutic drugs loaded with nanoparticles. Research has demonstrated that nanoparticles can enhance the absorption of medications like TMZ, a primary therapy for GBM, leading to improved effectiveness [23]. Nanoparticles are frequently designed with surface changes that enable them to bind to cell receptors that are excessively expressed in GBM cells [23]. This results in a higher concentration of drugs within the tumor [165]. The in vitro investigations consistently demonstrate that nanoparticles are more efficient than free drug formulations in reducing the viability of GBM cells [37].

Animal models have been used to conduct in vivo investigations that confirm the effectiveness of drug delivery systems using nanoparticles [21]. Animal models, namely mice models with xenografted GBM tumors, have played a crucial role in comprehending the pharmacokinetics, biodistribution, and therapeutic results of nanoparticles [2]. For instance, research investigations utilizing liposomal nanoparticles containing chemotherapeutic drugs have demonstrated a substantial decrease in tumor growth and an extended lifespan in treated mice, as compared with a control group [166]. Moreover, researchers have investigated the potential of nanoparticles, including gold nanoparticles, polymeric nanoparticles, and lipid-based nanoparticles, to traverse the BBB and provide medications directly to the site of brain tumors [167]. Although there have been achievements, there are still difficulties in completely using these discoveries in real-world medical environments. A major challenge is the intricate nature of the tumor microenvironment in humans, which can vary greatly from animal models, resulting in variations in how treatments are effective.

The outcomes of converting preclinical findings to clinical settings have been a combination of successes and obstacles. The successful use of nanoparticles in preclinical studies has generated greater interest in progressing these technologies to clinical trials. Nevertheless, the process of translating scientific findings from experimental settings to practical applications in healthcare is filled with difficulties, such as concerns over the ability to produce nanoparticles on a large scale, ensuring consistent and reliable outcomes, and the possibility of unintended impacts on non-target areas. Furthermore, the immunogenicity of specific nanoparticles is a substantial obstacle, as immune responses might result in fast removal from the bloodstream, thus diminishing therapeutic effectiveness. To manage these issues, a multidisciplinary strategy is necessary, one that involves cooperation between researchers, physicians, and regulatory agencies. The aim is to improve nanoparticle formulations and enhance their therapeutic use.

### 4.2. Clinical Trials Involving Nanoparticles in GBM Treatment

**Figure 5:**
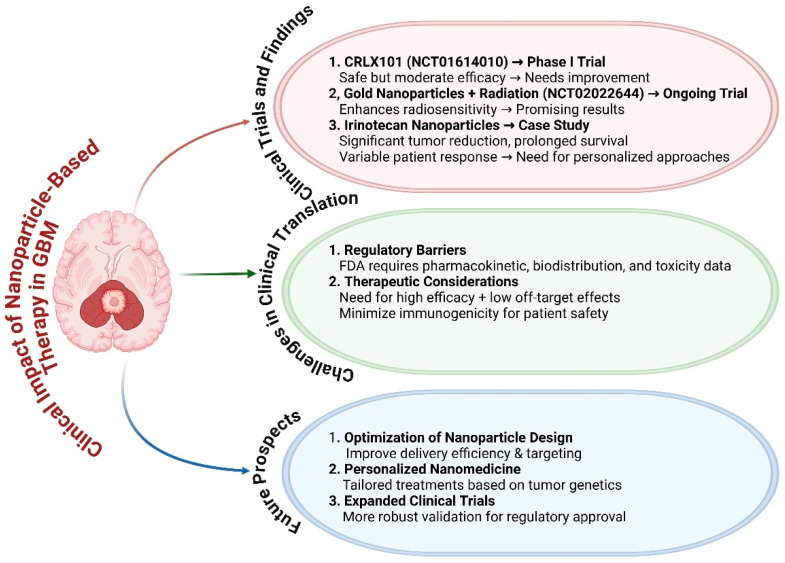
Clinical impact of nanoparticle-based therapy in GBM. The clinical impact of nanoparticle-based therapies in glioblastoma (GBM) treatment through three key sections. (1) Current clinical trials and findings: Summarizes trials such as those involving CRLX101 (NCT01614010), gold nanoparticles with radiation (NCT02022644), and irinotecan nanoparticles, highlighting their efficacy and challenges. (2) Challenges in clinical translation: Addresses regulatory hurdles, pharmacokinetics, toxicity, and immunogenicity concerns. (3) Future prospects: Emphasizes the optimization of nanoparticle design, personalized medicine, and expanded clinical trials. The figure was modified using the BioRender.com online commercial platform.

The successful transition from preclinical testing to clinical implementation is essential for the eventual incorporation of nanoparticles in GBM therapy. An examination of current and finished clinical trials indicates a burgeoning fascination in this field. Multiple clinical trials have been started to assess the safety and effectiveness of therapy based on nanoparticles in patients with GBM (Figure 5). An example of these is the Phase I clinical trial (NCT01614010) that examined the application of CRLX101, a nanoparticle-drug conjugate, in individuals who had recurrent GBM [168]. While the research showed that the treatment was safe, its effectiveness was only moderate, emphasizing the importance of further improvement [57]. A current clinical trial (NCT02022644) is investigating the application of gold nanoparticles in conjunction with radiation therapy for newly diagnosed GBM patients. Preliminary findings from this trial indicate an increased sensitivity to radiation, presenting a promising new approach for treating GBM [169]. Case reports and results from these experiments offer useful insights into the therapeutic capacity of nanoparticles [170]. A patient who had treatment with a nanoparticle-based formulation of irinotecan, a type of medication that inhibits topoisomerase, had a noteworthy outcome [171]. The patient experienced a substantial reduction in tumor size, which resulted in a longer period of life [88]. Nevertheless, these success stories are typically uncommon, as most patients do not experience the same degree of advantage. The diversity highlights the importance of personalized strategies in nanoparticle therapy, where treatment plans are customized according to unique patient attributes, such as tumor genetics and nanoparticle biodistribution profiles.

The clinical translation of nanoparticle-based therapeutics requires careful attention to regulatory considerations and safety profiles. Prior to granting clinical approval, the U.S. Food and Drug Administration (FDA) and other regulatory agencies mandate the submission of comprehensive safety data for nanoparticle formulations. This encompasses comprehensive evaluations of pharmacokinetics, biodistribution, and possible toxicity [172]. Nanoparticles must have both therapeutic effectiveness and little off-target effects, as well as low immunogenicity [173]. Although the requirements are strict, several formulations using nanoparticles have managed to meet these requirements and proceed to clinical trials. This is a significant achievement in the advancement of new treatments for GBM. Figure 5 highlights the promise of nanoparticles in GBM therapy, while there is a recognition of the need for further research to overcome translational barriers and improve clinical outcomes.

## 5. Challenges and Limitations of Nanoparticle-Mediated Drug Delivery in GBM Therapy

Despite the promising potential of nanoparticles in GBM therapy, several challenges and limitations hinder their clinical translation and efficacy. The use of nanoparticles for drug delivery has become a promising strategy by which to overcome the limitations of conventional therapies, enabling the target delivery of therapeutic molecules directly to the tumor site. However, significant challenges must be addressed to maximize their effectiveness in glioblastoma treatment.

### 5.1. Physiological Barriers

Nanoparticle-based drug delivery for GBM therapy faces multiple physiological barriers that limit its effectiveness and clinical translation. An important obstacle in using nanoparticles for drug delivery in glioblastoma therapy is the need to find ways to overcome the natural barriers within the BBB. While strategies such as receptor-mediated transcytosis and cell-penetrating peptides help improve transport, BBB integrity varies across different tumor regions, leading to inconsistent drug delivery [174]. Unlike the BBB, the blood–tumor barrier (BTB)in GBM is often leaky; however, its heterogeneous permeability can result in uneven drug distribution, limiting therapeutic efficacy [175]. Moreover, the tumor microenvironment (TME) of glioblastoma (GBM) is highly immunosuppressive and intricate, marked by hypoxia, acidic pH, and a dense extracellular matrix, which presents further challenges for nanoparticle infiltration and regulated medication release [176]. The reticuloendothelial system (RES) and mononuclear phagocyte system (MPS) are highly efficient at removing nanoparticles, thus decreasing their presence in the body and limiting their effectiveness as therapeutic agents [177]. Overcoming these physiological barriers requires innovative nanoparticle designs, including surface modifications, stimuli-responsive drug release, and strategies to transiently disrupt the BBB for enhanced drug delivery.

### 5.2. Scalability and Reproducibility in Manufacturing

The large-scale production of nanoparticles with consistent size, charge, and drug-loading capacity remains a major challenge, limiting their clinical application and regulatory approval [178]. Inconsistencies between batches can result in differences in the amount of medicine loaded, the rate at which it is released, and the capacity to target specific areas. These variations make it difficult to transition nanoparticle-based therapeutics from the laboratory to clinical settings. Stability and shelf-life issues pose significant challenges [58]. Nanoparticles must preserve their structural integrity and functional qualities throughout storage and transit, which can be influenced by variables such as temperature, pH, and light exposure [179]. The gradual deterioration or clumping together of nanoparticles over time can weaken their capacity to be used for medical treatment, which requires the creation of strong formulations that have improved stability [180]. Overcoming these challenges requires improvements in scalable production methods, automation, and standardized quality control protocols to guarantee the reproducibility and clinical efficacy of nanoparticle-mediated drug delivery systems for GBM treatment.

### 5.3. Toxicity and Off-Target Effects

Despite their potential for targeted drug delivery, nanoparticles pose concerns related to toxicity and unintended accumulation in non-target tissues, which can limit their clinical application in glioblastoma (GBM) therapy [181]. Nanoparticles could gather in tissues that are not the intended target, resulting in unintended consequences and toxicity throughout the body [182]. Nanoparticles, due to their small size and huge surface area, can interact with biological components in unanticipated manners, which can potentially lead to undesirable immunological responses, oxidative stress, and cytotoxicity [183]. The complete understanding of the biodistribution and removal mechanisms of nanoparticles is still lacking, which gives rise to uncertainties regarding their potential long-term effects on human health [184]. Non-biodegradable nanoparticles provide concerns of prolonged accumulation in tissues, potentially resulting in chronic toxicity. Facilitating effective elimination via renal or hepatic routes is essential for reducing unwanted effects [185]. Some nanoparticles, particularly metallic or polymeric types, may trigger inflammatory responses, oxidative stress, or immune activation, leading to unintended side effects and reduced therapeutic efficiency. Addressing these challenges requires the creation of biocompatible, biodegradable, and targeted nanoparticles with improved safety profiles. Surface changes, regulated drug release mechanisms, and thorough preclinical toxicity evaluations are crucial for enhancing the therapeutic efficacy of nanoparticle-mediated GBM treatment.

## 6. Future Perspectives of Nanoparticle-Mediated Drug Delivery in GBM Therapy

**Figure 6:**
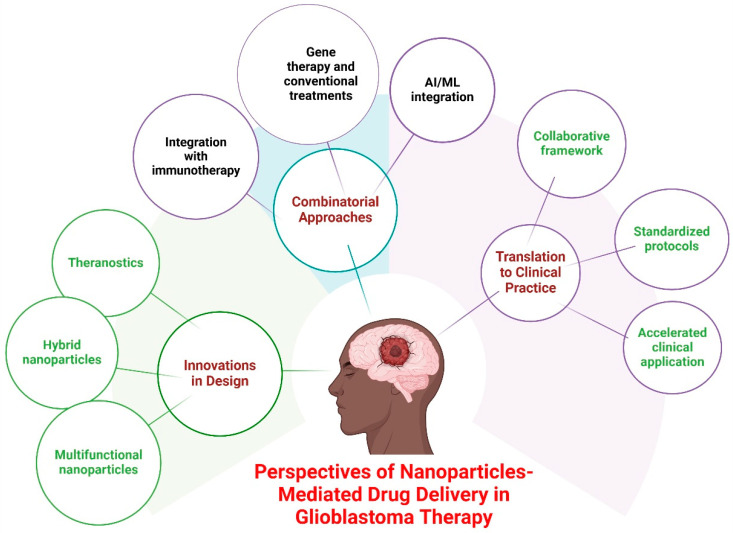
A conceptual diagram illustrates the future perspectives of nanoparticle-mediated drug delivery for glioblastoma therapy. This summary and illustration encapsulate the transformative potential of nanoparticles in glioblastoma therapy, emphasizing advancements in design, emerging therapeutic strategies, and the critical need for effective clinical translation. The figure was modified using the BioRender.com online commercial platform.

Future research and technology improvements are anticipated to overcome current limitations and improve the clinical usefulness of these novel drug delivery methods. As research advances, multidisciplinary collaborations across nanotechnology, oncology, and computational science will be essential for enhancing nanoparticle-mediated drug delivery for more effective and practical GBM treatments. A summary of future perspectives of nanoparticle-mediated drug delivery in glioblastoma therapy is presented in Figure 6.

### 6.1. Innovations in Nanoparticle Design

Improvements in nanoparticle design are essential for optimizing drug delivery efficacy, reducing off-target effects, and surmounting physiological challenges in GBM treatment. Future advancements in nanoparticle engineering concentrate on enhancing targeting, drug release, and biocompatibility [79]. The integration of multiple functional components within a single nanoparticle, such as drug-loaded cores with imaging agents or immune-modulating molecules, can facilitate combination therapy and real-time monitoring of treatment efficacy [186]. The production of biomimetic nanoparticles, including those enveloped in cell membranes or originating from natural polymers, augments biocompatibility, diminishes immunogenicity, and boosts BBB penetration [187]. In addition, the emergence of personalized medicine has introduced the notion of theragnostics, which involves the integration of therapy and diagnostics [188]. Nanoparticles can be customized to match the precise molecular characteristics of an individual’s tumor, allowing for personalized therapy approaches that optimize effectiveness while avoiding adverse reactions [189]. Theragnostic nanoparticles enable the simultaneous monitoring of treatment response in real-time, providing a dynamic approach to glioblastoma therapy [190]. Utilizing these advancements, advanced nanoparticle-based therapeutics may substantially boost drug delivery efficiency, improve patient outcomes, and facilitate precision medicine strategies in the treatment of GBM.

### 6.2. Emerging Therapies and Combinatorial Approaches

The combination of nanoparticles with novel medicines and combinatorial treatment approaches provides a promising opportunity to augment the effectiveness of GBM treatment. These approaches seek to utilize synergistic effects to surmount therapeutic resistance and enhance patient outcomes [23]. Nanoparticles can be designed to deliver immune checkpoint inhibitors, cytokines, or tumor-associated antigens, thereby augmenting the immune system’s capacity to identify and eradicate GBM cells. Immune-modulating nanoparticles may assist in reprogramming the immunosuppressive tumor microenvironment. Nanoparticles can serve as radiosensitizers, enhancing the effects of radiotherapy by increasing DNA damage in tumor cells while reducing radiation exposure to healthy tissues. Gold and gadolinium-based nanoparticles have shown promise in preclinical studies. Moreover, the utilization of artificial intelligence (AI) and machine learning (ML) to enhance the process of designing and delivering nanoparticles is becoming increasingly popular. AI and ML have the capability to examine large datasets to forecast the most efficient compositions of nanoparticles, enhance medication loading, and customize delivery methods for patients. Utilizing data-driven methods can expedite the advancement of nanoparticles for future applications and enhance their effectiveness in clinical settings. Integrating nanoparticle technology with developing medicines may enhance future GBM treatment options by surmounting existing therapeutic limits, increasing drug efficacy, and prolonging patient survival. Additional research and clinical validation will be crucial for converting these advancements into viable therapeutic applications.

### 6.3. Translation from Bench to Bedside

Despite substantial advancements in nanoparticle research for glioblastoma treatment, the progression from preclinical investigations to clinical implementations continues to pose a considerable hurdle. Multiple critical criteria must be considered to enable the effective implementation of nanoparticle-based drug delivery systems in standard clinical practice. Nanoparticle-based medicines must comply with rigorous regulatory standards concerning safety, effectiveness, and reproducibility. Implementing established processes for nanoparticle characterization, quality assurance, and large-scale production is crucial for obtaining regulatory approval from authorities like the FDA and EMA. Numerous nanoparticle formulations have shown encouraging outcomes in preclinical models yet faltered in clinical trials owing to disparities in tumor biology, medication metabolism, and patient variability. Executing meticulously designed clinical trials with rigorous patient stratification criteria is essential for establishing efficacy in human subjects. The mass manufacture of nanoparticles with uniform size, shape, and drug-loading efficacy is essential for commercial feasibility. Progress in automation, microfluidics, and GMP-compliant manufacturing techniques will enhance clinical translation. Comprehending the long-term biodistribution, clearance mechanisms, and potential toxicity of nanoparticles is essential for patient safety. Comprehensive pharmacokinetic and toxicological investigations are required to assess the chronic effects of nanoparticle accumulation in various organs. Effective translation necessitates cooperation among nanotechnologists, oncologists, regulatory specialists, and the pharmaceutical sector. Collaborative work across multiple disciplines will expedite the advancement of clinically applicable nanoparticle-based therapeutics. By overcoming these limitations, nanoparticle-mediated drug delivery systems could transform GBM treatment, providing more efficient and focused therapeutic alternatives for patients. Continued progress in translational research and clinical validation will be crucial for delivering these breakthrough treatments to patients.

## 7. Conclusions

Nanoparticles have become a potential method for treating GBM because of their distinct physical and chemical features, which allow for targeted medication administration and better treatment results [191]. The main advantages encompass improved drug solubility, regulated release, and the capacity to traverse the BBB, a significant obstacle in the treatment of GBM [192]. The process of attaching targeting ligands to nanoparticles enables the specific delivery of the nanoparticles to tumor cells, thus decreasing unintended effects on non-target cells and reducing overall toxicity in the body. In addition, nanoparticles can be designed to release therapeutic substances in response to stimuli, like changes in pH or the presence of enzymes, thus enhancing the effectiveness of treatment. Despite these benefits, there are still numerous obstacles that remain. The diversity of GBM and the existence of a highly varied tumor microenvironment make it difficult to design nanoparticle-based therapies that are uniformly successful. Nanoparticles have the capacity to accumulate in tissues that are not intended to be targeted, which can result in unexpected adverse effects. Moreover, the ability to increase the size of nanoparticle production and the intricate regulatory challenges linked to their clinical application continue to be major obstacles. It is essential to maintain uniform quality and safety of nanoparticles in various manufacturing batches to effectively incorporate them into clinical practice.

Extracellular vesicles (EVs), such as exosomes, have been identified as effective natural carriers for drug delivery in the treatment of glioblastoma. Nanosized vesicles, secreted by diverse cell types, can traverse the BBB bidirectionally, enabling targeted drug delivery to glioblastoma cells. EVs demonstrate lower immunogenicity compared with synthetic nanoparticles, thereby minimizing the potential for adverse immune reactions. Furthermore, they can be designed to encapsulate therapeutic agents such as chemotherapeutics, siRNA, and miRNA, thereby improving drug bioavailability and efficacy. EVs exhibit intrinsic targeting properties and biocompatibility, making them a promising minimally invasive approach for glioblastoma treatment, thus necessitating additional clinical research.

The potential of nanoparticle-mediated medication delivery in GBM therapy is highly promising. Current research endeavors to surpass current constraints by creating versatile nanoparticles that integrate imaging, diagnostics, and therapy into a unified platform. Progress in nanotechnology and an improved comprehension of GBM biology are anticipated to stimulate advancements in nanoparticle development, resulting in enhanced and tailored treatment approaches. By incorporating nanoparticles into new treatment techniques like immunotherapy and gene editing, their effectiveness can be significantly augmented. To summarize, despite the existence of obstacles, the ongoing development of nanoparticle technology and its use in GBM therapy present a promising prospect for enhancing patient results. Nanoparticles, via continuous research and clinical trials, have the potential to revolutionize the treatment approaches for GBM, ultimately enhancing survival rates and improving the quality of life for patients.

## Figures and Tables

**Table 1:** Different nanoparticles and their delivery for the treatment of GBM therapy.

Number	Nanoparticle	Size (nm)	Delivery System	Molecular Action in GBM Therapy	References
1	Liposomes	50–200	Lipid-based	Encapsulate and deliver chemotherapeutics, improve stability and reduce toxicity	[97]
2	Solid lipid nanoparticles (SLNs)	50–1000	Lipid-based	Encapsulate drugs, control release, target GBM cells	[98]
3	Polymeric micelles	10–100	Amphiphilic block copolymers	Improve drug solubility, targeted delivery, enhance cellular uptake	[99]
4	Gold nanoparticles	15–50	Metal-based	Enhance radiotherapy effects, targeted drug delivery	[100]
5	Magnetic nanoparticles	10–100	Metal-based, magnetic	Targeted drug delivery using external magnetic fields, imaging	[101]
6	Quantum dots	2–10	Semiconductor	Imaging, drug delivery, fluorescence-based targeting	[93]
7	Dendrimers	1–10	Polymeric	Targeted delivery, multiple drug loading sites	[102]
8	Carbon nanotubes	1–100	Carbon-based	Drug delivery, imaging, enhance therapeutic efficacy	[103]
9	Mesoporous silica nanoparticles	50–300	Silica-based	Drug loading, controlled release, targeted delivery	[104]
10	Hydrogels	10–1000	Hydrogel-based	Drug delivery, tissue engineering, localized therapy	[105]
11	Nanospheres	50–500	Polymeric, metal, or silica-based	Encapsulate and release drugs, enhance cellular uptake	[106]
12	Nanosuspensions	50–1000	Suspension of nanoparticles in liquid	Improve drug solubility, bioavailability, targeted delivery	[107]
13	Lipid-core nanocapsules	50–200	Core–shell lipid-based	Protect drug, enhance bioavailability, targeted delivery	[108]
14	PLGA nanoparticles	100–1000	Biodegradable polymeric	Controlled drug release, biocompatibility, targeting	[109]
15	Iron oxide nanoparticles	1–100	Magnetic, metal-based	Targeted delivery, imaging, therapy enhancement	[110]
16	Polyethylene glycol (PEG)-coated nanoparticles	50–200	Polymeric, with PEG coating	Enhanced circulation time, reduced immune recognition	[111]
17	Poly(lactic-co-glycolic acid) (PLGA) nanoparticles	100–500	Biodegradable polymeric	Controlled release, biocompatibility, targeted therapy	[112]
18	Calcium phosphate nanoparticles	50–200	Mineral-based	Controlled drug release, gene delivery	[113]
19	Bioresponsive nanoparticles	50–200	Various materials, responsive to stimuli	Controlled release in response to physiological conditions	[114]
20	Hybrid nanoparticles	50–300	Combination of different materials	Multi-functional drug delivery, enhanced targeting	[115]

**Table 2:** Recently used preclinical and clinical studies of nanoparticle-mediated drug delivery for GBM therapy.

Nanoparticle	Size	Molecular Action	Preclinical Studies	Clinical Studies	References
Liposomes	50–200 nm	Encapsulation of drugs; improved delivery and cellular uptake	Significant tumor reduction and improved survival in animal models	Phase I/II trials with some success in enhancing drug delivery	[150]
Gold nanoparticles	10–100 nm	Enhanced drug delivery; photothermal therapy	Effective in reducing tumor volume in animal studies	Limited clinical trials: early results are promising	[151]
Magnetic nanoparticles	10–50 nm	Targeted delivery using magnetic fields; localized hyperthermia	Effective targeting and reduction in glioblastoma models	Ongoing trials evaluating safety and efficacy	[152]
Silica nanoparticles	20–100 nm	Targeted drug delivery; imaging and diagnostics	Reduction in tumor growth in animal models	Not yet in clinical trials, primarily used for preclinical research	[153]
Polymeric nanoparticles	50–200 nm	Controlled release of drugs; targeting specific cells	Significant tumor suppression in animal studies	Early-phase trials show promise in improving delivery	[154]
Dendrimers	4–10 nm	High drug payload capacity; targeted delivery	Effective in reducing glioblastoma in preclinical studies	Phase I trials in progress; potential for improved targeting	[155]
Quantum dots	2–10 nm	Imaging and tracking; targeted drug delivery	Tumor imaging and drug delivery enhancements in preclinical models	Not yet in clinical trials, primarily used for research	[156]
Carbon nanotubes	1–100 nm	Enhanced drug delivery; potential for photothermal therapy	Effective in animal models for targeted delivery and treatment	Early research with potential for future clinical trials	[157]
Metal–organic frameworks (MOFs)	50–100 nm	Drug delivery; imaging and therapy	Effective drug delivery and imaging in preclinical studies	Initial clinical trials are ongoing	[158]
Hydrogel nanoparticles	100–200 nm	Controlled drug release; tissue engineering	Effective in targeted delivery and reduced tumor growth in animals	Early-phase studies; potential for improved drug delivery	[159]
Cerium oxide nanoparticles	5–50 nm	Antioxidant properties; targeted drug delivery	Reduction in glioblastoma progression in animal models	Limited clinical data: preclinical research is ongoing	[160]
Chitosan nanoparticles	50–200 nm	Drug encapsulation; enhanced permeability and retention effect	Effective drug delivery and reduced tumor volume in preclinical studies	Early research with potential for future trials	[161]
Polyethylene glycol (PEG)-coated nanoparticles	50–200 nm	Improved circulation time; reduced immune response	Enhanced delivery and reduced tumor growth in preclinical models	Early-phase clinical trials show improved efficacy	[162]
Nanospheres	10–200 nm	Targeted delivery; high surface area for drug loading	Effective in animal models; enhanced drug delivery	Not widely in clinical trials; research ongoing	[163]
Micelles	10–100 nm	Encapsulation of hydrophobic drugs; targeted delivery	Significant reduction in tumor growth in animal studies	Clinical trials show promise in enhancing drug delivery	[164]

## Abbreviations

NPs	Nanoparticles
GBM	Glioblastoma multiforme
TMZ	temozolomide
BBB	Blood–brain barrier
EPR	Enhanced permeability and retention
SLNs	Solid lipid nanoparticles
CNS	Central nervous system
PLGA	Poly(lactic-co-glycolic acid)
AuNPs	Gold nanoparticles
IONPs	Nanoparticles of iron oxide
MRI	Magnetic resonance imaging
CNTs	Carbon nanotubes
QDs	Quantum dots
RES	Reticuloendothelial system
CPPs	cell-penetrating peptides
GPCRs	G protein-coupled receptors
ECM	Extracellular matrix
EVs	Extracellular vesicles
